# Dependence of tropical cyclone seeds and climate sensitivity on tropical cloud response

**DOI:** 10.1126/sciadv.adi2779

**Published:** 2024-09-11

**Authors:** Tsung-Lin Hsieh, Gabriel A. Vecchi, Chenggong Wang, Wenchang Yang, Bosong Zhang, Brian J. Soden

**Affiliations:** ^1^ Program in Atmospheric and Oceanic Sciences, Princeton University, 300 Forrestal Rd, Princeton, NJ 08544, USA.; ^2^ Department of Geosciences, Princeton University, Guyot Hall, Princeton University, Princeton, NJ 08544, USA.; ^3^ Rosenstiel School of Marine, Atmospheric, and Earth Science, University of Miami, 4600 Rickenbacker Causeway, Miami, FL 33149, USA.

## Abstract

Projections of future tropical cyclone frequency are uncertain, ranging from a slight increase to a considerable decrease according to climate models. Estimation of how much the Earth’s surface temperature warms in response to greenhouse gas increase, quantified by effective climate sensitivity, is also uncertain. These two uncertainties have historically been studied independently as they concern different scales: One quantifies the extreme weather and the other the mean climate. Here, we show that these two uncertainties are not independent and are both influenced by the response of tropical clouds to warming. Across climate models, we show an anticorrelation between shortwave cloud radiative feedback and changes in the frequency of seed vortices, a prevalent type of tropical cyclone precursors. We further show an anticorrelation between effective climate sensitivity and tropical cyclone frequency changes, suggesting that global tropical cyclone frequency tends to decrease more substantially in models with larger temperature increase.

## Teaser

Tropical cyclone and global warming projections are uncertain, but the combination of these effects is constrained by theory.

## INTRODUCTION

The future change in tropical cyclone (TC) frequency remains uncertain (*1*). Across global climate models, the changes in TC frequency between the historical and future simulations range from decreasing by ∼20% to increasing by ∼10% per 2 K warming (*1*). Because all TC impacts are connected to TC frequency, this wide range of uncertainty creates challenges for downstream applications such as the estimation of TC hazards and risks and, in turn, difficulties in climate adaptation.

While many climate models have simulated a decrease in TC frequency with warming, one physically plausible scenario in which TC frequency increases is simulated by a model known as the High-Resolution Forecast-Oriented Low Ocean Resolution (HiFLOR) model (*2*). Despite being on the upper limit of the uncertainty range, this model has compared well against historical TC data (*3*), and there has been no physical evidence that can rule out the plausibility of its TC projection (*4*). In addition, the increase in TC frequency in this model agrees qualitatively with results given by an independent TC downscaling estimation (*5*). It is not yet possible to eliminate the potential for future TC increase by existing theories and observations (*6*).

The future climate simulated by the HiFLOR model has less warming in response to increasing greenhouse gas concentrations [i.e., a low effective climate sensitivity (ECS)] than other global climate models due to a negative cloud radiative feedback, which dampens the climate response to increasing greenhouse gas concentration (*7*). Like the projections of TC frequency, the magnitude of cloud radiative feedback is also uncertain and varies widely among global climate models. The uncertainty in cloud radiative feedback has long been a primary source of uncertainty for the ECS (*8*, *9*).

We hypothesize that the uncertainties in ECS and TC projection across climate models are not independent. Satellite observations have shown that clouds associated with TCs influence the radiative fluxes, which lead to net cooling of the Earth system (*10*). Conversely, model experiments have shown that the climatological radiation anomalies can alter the TC frequency through a change in atmospheric circulation (*11*). In other words, the radiative effect of clouds and the associated changes in atmospheric circulation may be a common cause for the uncertainties in future global mean temperature increase and TC frequency change.

Studies of TC frequency and ECS have historically been performed separately due in part to the limitation of computational technology. To simulate the TC frequency response to warming, it is preferable to use global models with a 50-km or finer horizontal grid spacing to generate realistic geographical distribution of TCs. In addition, because TC occurrences are rare, a simulation length of decades is required to separate the climate signal from atmospheric variability (*12*). In contrast, models used to study ECS are more sophisticated and capture interactions between the atmosphere, land, ocean, and ice processes. These models are therefore typically coarser in resolution (≈100 km or more) to allow for longer and more sophisticated simulations as well as multiple ensemble members (*13*). Furthermore, to capture the uncertainty in TC projections and ECS, a diverse set of models is required to sample the plausible range of model parameters and designs, which demands even more computational resources.

To bridge the gap between the two classes of models used to study TC projections and ECS, we use a recently developed downscaling theory expressed as the seed propensity index (SPI) (*14*). The index has been shown to capture the wide range of TC projections across high-resolution models (*4*) based on climatological mean variables that are resolved in coarse-resolution climate models. The index quantifies the frequency of seed vortices, which are TC precursors and are known to be a root cause of the model spread in TC projections (*15*). Therefore, the downscaling theory enables a physical comparison between low- and high-resolution climate models.

We analyze three complementary sets of simulations, each having its own strengths and limitations in simulating changes in the global mean surface temperature and TCs. The first set of simulations uses models in Coupled Model Intercomparison Project Phase 6 (CMIP6) (*13*), which are used to estimate the ECS and large-scale cloud response patterns. The second and third sets of simulations use high-resolution atmospheric models (AMs) with prescribed sea surface temperature (SST), which generate detailed distributions of cloud feedback and TC response to SST perturbations. The second set of simulations addresses the influence of uniform SST increase, while the third set of simulations addresses the effect of SST perturbation patterns by calculating the Green’s function. By combining theory and modeling, we seek to understand the physical mechanisms and conditions in which TC projections and the ECS may be commonly dependent on the tropical cloud response.

## RESULTS

### Correlation across climate models

**Fig. 1.**
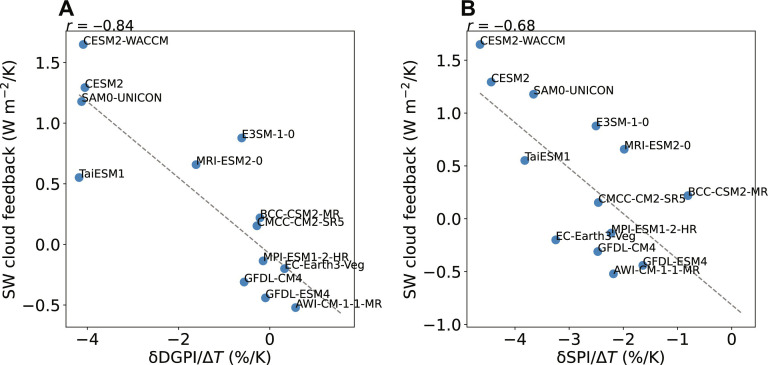
Correlation across CMIP6 coupled climate models. The shortwave cloud feedback versus the percentage changes in (**A**) the DGPI (δDGPI) and (**B**) the SPI (δSPI) per Kelvin warming. CMIP6 models having a horizontal grid spacing of 200 km or coarser are ignored due to the poorly resolved tropical cloud patterns. All quantities are averaged over the tropical Northern Hemisphere ocean.

To investigate the response of clouds and TCs to the increase in greenhouse gas concentration, we examine CMIP6 models with coupled atmosphere and ocean dynamics. Two experiments are analyzed for each model: a control experiment with greenhouse gases and aerosols under the preindustrial condition and a perturbation experiment forced by an abrupt quadrupling of atmospheric carbon dioxide (CO_2_) concentration. Elements in the Earth system adjust with the surface temperature in response to forcing, quantified by the climate feedback parameter (see Materials and Methods). In particular, the shortwave cloud feedback measures how much shortwave radiation the Earth gains due to the change in clouds per Kelvin of global mean surface warming, shown in the vertical axes of Fig. 1. It is one of the largest sources of uncertainty in the intermodel spread in the ECS (*9*).

Because TCs are not well resolved in these CMIP6 models, we measure the dynamical genesis potential index (DGPI) (*16*) as a proxy for TC frequency (see Materials and Methods). This index is known to capture the wide range of global mean TC response across models (*16*) thanks to its inclusion of vertical velocity as a large-scale environmental predictor. The percentage change in DGPI is measured between the preindustrial control and the perturbed climates after 100 years of simulation.

In addition to the DGPI, we measure the SPI, which has a more straightforward physical interpretation while also capturing the model spread in TC frequency projections (*4*). The SPI is designed to parameterize the frequency of seed vortices, which may evolve into TCs. Over plausible future climates, the model spread in TC frequency is known to follow the model spread in seed frequency (*4*, *15*). The SPI provides an additional advantage that it has a physically transparent, analytical connection with radiation (*11*), while other TC proxies have unknown relationships with radiation.

Figure 1A suggests that the model spread in shortwave cloud feedback is correlated with the TC proxy, namely, DGPI, particularly over the tropical Northern Hemisphere ocean. The correlation indicates that models with stronger TC reduction tend to have a more positive shortwave cloud feedback, meaning that the Earth absorbs more solar radiation due to the change in clouds. This correlation is consistent with the correlation between the SPI and shortwave cloud feedback (Fig. 1B). The latter can be interpreted as a result of the coupling between deep convection and the shortwave cloud feedback: When deep convection becomes more active, more shallow clouds outside the convective region tend to form due to the enhanced stability, reflecting more solar radiation and creating a negative shortwave cloud feedback (*17*–*20*). At the same time, when deep convection becomes more active, large-scale vertical velocity increases (*21*), and the SPI increases where background vorticity is sufficient. That is, the seed response and the shortwave cloud feedback are both dependent on the deep convection response to warming under the following conditions.

The correlation applies specifically when averaged over the tropical Northern Hemisphere ocean, shown in Fig. 1. Over land without sufficient moisture supply, the activity of deep convection is irrelevant to TC formation. The South Pacific and South Atlantic are also unfavorable for TC formation regardless of the change in clouds. The change in clouds on the equator is irrelevant for TC genesis due to the lack of background vorticity, but we found that their influence is relatively minor, and the correlation holds across the entire tropical Northern Hemisphere ocean.

### Response to uniform warming

In CMIP6 models, the shortwave cloud feedback (λ) and SPI response (δSPI/Δ*T*) are influenced by several factors, including the SST perturbation (ΔSST) and the formulation of the AM

$$\lambda =f_{1}\left(\text{ΔSST},\text{AM}\right)$$ (1A)

$$\text{δSPI}/\Delta T=f_{2}\left(\text{ΔSST},\text{AM}\right)$$ (1B)

We perturb these two factors separately and examine how the correlation between λ and (δSPI/Δ*T*) holds across the phase space. This section concerns *f_i_*(AM)|_ΔSST_, while the following section concerns *f_i_*(ΔSST)|_AM_.

We use the prescribed SST technique to accelerate computation by simulating only the atmospheric dynamics, which allows for enhanced resolution with 50- and 25-km horizontal grid spacings that better resolve the TC distribution than CMIP6 models with 100-km grid spacing. As a result, we are able to track and count TCs simulated by these models. Three AMs are used, and two experiments are performed for each model: one driven by the climatological SST annual cycle and the other by an additional 2 K of uniform sea surface warming. Here, we focus on uniform warming as the response to uniform ΔSST is a more important source of model spread than to patterned ΔSST (*4*). We prescribed identical SST fields to all three AMs.

**Fig. 2.**
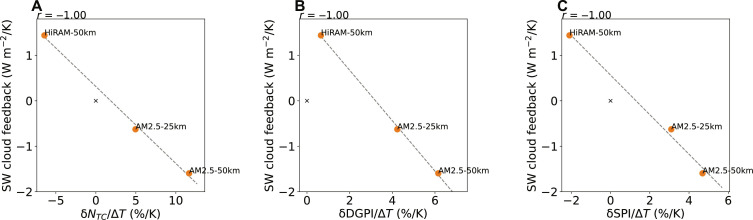
Correlation across uniform warming experiments in AMs. The shortwave cloud feedback versus the percentage changes in (**A**) the frequency of explicitly simulated TCs (δ*N*_TC_), (**B**) the DGPI (δDGPI), and (**C**) the SPI (δSPI) per Kelvin warming, averaged over all months over the tropical Northern Hemisphere ocean.

Figure 2 shows that the shortwave cloud feedback is negatively correlated with the TC response, the DGPI response, and the SPI response, similar to results from the CMIP6 coupled models in Fig. 1. The SPI response captures the TC frequency decrease in the High Resolution Atmospheric Model (HiRAM) and the increase in both Atmospheric Model version 2.5 (AM2.5) resolutions, while the DGPI response generally has more positive values than the SPI response. Although large-scale TC proxies are not expected to quantitatively match the explicitly simulated TC frequency (*22*), we find that the SPI is useful for identifying the large-scale drivers of the model spread.

**Fig. 3.**
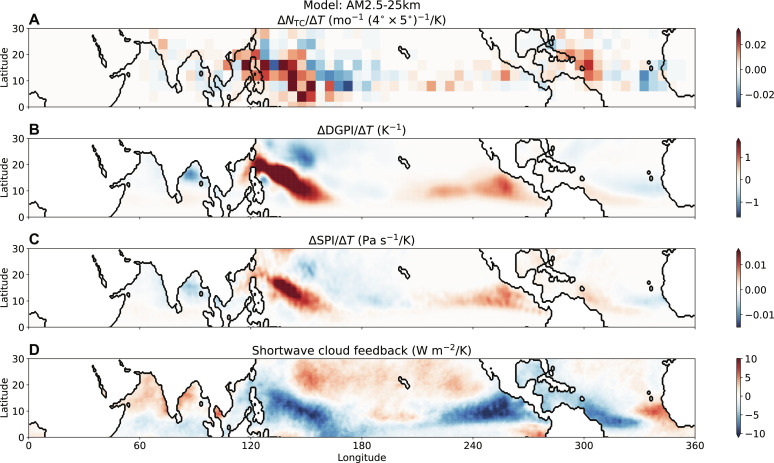
Spatial distribution of response to uniform warming. (**A**) Change in TC genesis frequency per Kelvin warming, (**B**) the change in DGPI per Kelvin warming, (**C**) the change in SPI per Kelvin warming, and (**D**) the shortwave cloud feedback in the AM2.5-25 km model. In these maps, the quantities are averaged from June to November, the Northern Hemisphere TC season. mo, month.

We further examined the spatial maps of these fields, shown in Fig. 3 for our highest-resolution model, AM2.5 with 25-km resolution (AM2.5-25 km), and for the other two models in figs. S1 and S2. The DGPI and SPI response patterns resemble a smoothed version of the discrete TC genesis counts as the indices represent the primary large-scale drivers of TC genesis. They are predominantly influenced by regions with large-scale climatological ascent away from the equator and are zero in descending regions. While the shortwave cloud feedback is nonzero in ascending and descending regions, the tropical mean is also primarily influenced by the ascending regions. The results suggest that the correlation between TC response and shortwave cloud feedback holds not only across CMIP6 models but also across higher-resolution models in which TCs are simulated explicitly.

### Response to SST patterns

We seek to understand if the SST perturbation pattern influences the correlation between TC response and shortwave cloud feedback and which region of relative warming is the most important. That is, we investigate *f_i_*(ΔSST)|_AM_ defined earlier. To efficiently sample the infinite possibility of SST perturbation patterns, we calculate the Green’s function for localized SST increase with a small amplitude. The response to a general SST perturbation pattern is to a good approximation the linear superposition of the Green’s function (*23*, *24*). This technique has been used to study the cloud feedback (*25*), and here we further calculated the Green’s function of the TC and seed proxies.

**Fig. 4.**
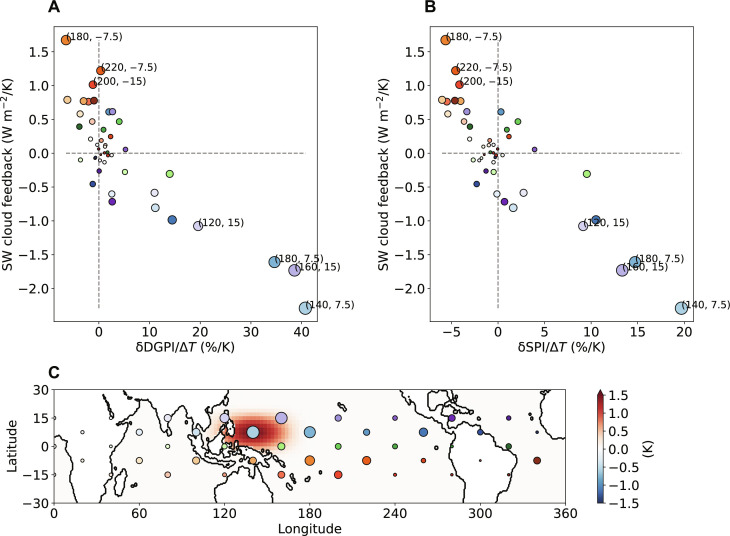
Correlation across localized SST perturbations. The shortwave cloud feedback versus the percentage changes in (**A**) the DGPI (δDGPI) and (**B**) the SPI (δSPI), averaged over the tropical Northern Hemisphere ocean. Here, Δ*T* represents the magnitude of the SST perturbation (1.5 K), rather than the global mean surface warming as in the other figures. Numbers in parentheses indicate the longitude and latitude. (**C**) Example of ΔSST centered at 140° longitude and 7.5° latitude. The circles indicate the centers of the perturbed SST patches. Colder colors represent more northward latitudes, and darker shades represent more eastward longitudes. The radii of the circles represent the importance in driving the correlation, i.e., the distance to origin in (A).

Figure 4 (A and B) shows that the shortwave cloud feedback is negatively correlated with the TC and seed proxies in response to localized SST perturbations, consistent with Figs. 1 and 2. To cover the entire tropical ocean, we perturbed the SST around 45 locations shown in Fig. 4C in an AM with 100-km resolution for computational feasibility required by the large number of simulations. The magnitude of the perturbation is 1.5 K and is identical for all 45 locations.

The lower right corner in Fig. 4 (A and B) suggests that warming in the western North Pacific tends to generate a negative shortwave cloud feedback when averaged over the tropical Northern Hemisphere ocean. At the same time, these perturbations lead to increases in seeds and TCs. In contrast, the upper left corner represents the response to central South Pacific warming, which causes an equatorward shift of the intertropical convergence zone that decreases the seed and TC frequency while increasing the shortwave cloud feedback. The Green’s function shows that SST perturbation in the western North Pacific is the most important driver for the correlation between the TC response and shortwave cloud feedback. In other words, the correlation is agnostic to SST response patterns as long as the uncertainty is dominated by the western or southern Pacific SST.

### Implications on the effective climate sensitivity

**Fig. 5.**
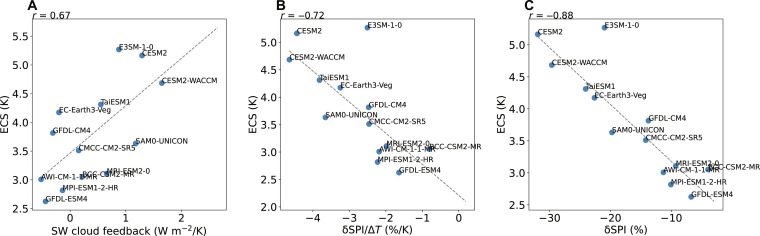
Climate sensitivity and TC seeds across CMIP6 coupled models. The global mean ECS versus (**A**) the shortwave cloud feedback, (**B**) the percentage change in SPI (δSPI) per Kelvin warming, and (**C**) the percentage change in SPI (δSPI). Quantities on the horizontal axes are averaged over the tropical Northern Hemisphere ocean.

We have focused on the tropical Northern Hemisphere ocean in the analysis, but the ECS is a global mean quantity. While the global mean shortwave cloud feedback is known to correlate with the ECS (*26*), here it is important to verify that shortwave cloud feedback averaged over the tropical Northern Hemisphere ocean is also correlated with the global mean ECS. Figure 5A suggests that tropical clouds over the Northern Hemisphere ocean contribute appreciably to the model spread, despite not being the sole source of uncertainty (*9*).

Similarly, Fig. 5B shows that the SPI response averaged over the tropical Northern Hemisphere ocean is correlated with the global mean ECS. This correlation reflects the combination of the positive correlation between the ECS and shortwave cloud feedback (Fig. 5A) and the negative correlation between the shortwave cloud feedback and SPI (Fig. 1B). We focus on the SPI because it has a more robust physical connection with deep convection than the DGPI does. It is possible that other factors may also contribute to this correlation, such as the relationship between the ECS and the interhemispheric asymmetry (*26*). Nevertheless, our results show that the correlation between the shortwave cloud feedback and SPI response is a robust and physically consistent driver for the correlation between the ECS and SPI response.

Last, we show the total percentage change in the SPI, rather than the percentage change per Kelvin as in previous figures. The latter is a more fundamental physical property of the climate system, but the former is a more societally relevant measure. Figure 5C suggests that the ECS is highly correlated with the percentage change in the SPI, with a linear correlation of *r* = −0.88. This strong correlation suggests that models that warm more substantially (higher ECS) tend to have more rapid decrease in the SPI and potentially in TC frequency.

## DISCUSSION

**Fig. 6.**
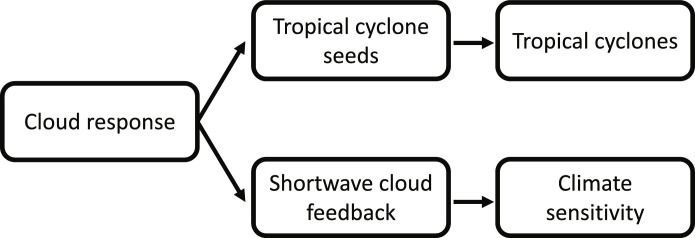
Direction of causality. The response of tropical clouds, which varies across models, influences both TC seeds and shortwave cloud feedback. This common dependency leads to a correlation between the TC response and climate sensitivity.

We have established a chain of physical links (Fig. 6) that correlates the future increase in global mean surface temperature (quantified by the ECS) with the change in TC seed frequency (estimated by the SPI). The starting point is the variation in deep convection response, which is highly uncertain across models. When deep convection becomes less active, especially in the Western Pacific, two consequences follow: the first is a positive shortwave cloud feedback and the second is a decreased SPI. Models with a more positive shortwave cloud feedback tend to warm faster, leading to a higher ECS. At the same time, a more substantial decrease in the SPI likely leads to a reduction in TC frequency. Note that we do not interpret this correlation as a feedback between the TC frequency and ECS but rather a common dependence on the tropical cloud response.

Although current climate models do not generate consistent future projections of the TC frequency or ECS, our work uncovered a fundamental constraint underlying these findings. Our results reiterate the importance of constraining the cloud response in models, which remains an active area of research (*9*). In climate models, this uncertainty is sometimes encoded in the parameterization of convective detrainment efficiency: If a model is designed such that the detrainment efficiency decreases with warming, then it tends to show a more positive cloud feedback (*27*). In addition, the cloud response is sensitive to the pattern of SST response, which remains unconstrained by models (*28*). In any case, our results suggest that uncertainties in TC projections and ECS are likely correlated as long as the uncertainty in future SST response is dominated by the Pacific Ocean.

The fact that uncertainty in TC frequency is closely tied to uncertainty in seed frequency is key to linking TC projections to climate change. TCs are rare, extreme events whose dependence on the mean climate is difficult to constrain (*29*). On the other hand, seeds are more abundant and have a more straightforward connection with the mean circulation. We have shown in models that TC seeds are intimately related to the cloud feedback pattern. We have also identified the limitation of this relationship: It does not hold in areas with minimal TC seed formation, including land surfaces, low latitudes with small background vorticity, and parts of the Southern Hemisphere. Furthermore, the transition from seeds to TCs may be influenced by environmental factors that are not directly related to cloud radiation. Nevertheless, given the strong correlation between the ECS and SPI (Fig. 5C), we suggest that ECS and TC projections are likely not independent, at least in climate models.

This constraint has important consequences for the globally aggregated TC hazards. The change in TC frequency directly influences the probability of TC landfall, and the location of TC seed formation plays a critical role in determining the TC trajectory (*12*). At the same time, the amount of ocean warming determines the rate of increase in TC intensity and the associated precipitation (*30*). A negative correlation between the TC seed response and shortwave cloud feedback suggests that, for a certain radiative forcing perturbation, the Earth is unlikely to have both a very low (high) climate sensitivity and a large decrease (increase) in global TC frequency with warming.

## MATERIALS AND METHODS

### Dynamical genesis potential index

The DGPI (*16*) is a proxy for TC frequency, defined as

$$\begin{array}{c} \text{DGPI}={\left(2.0+0.1V_{s}\right)}^{-1.7}{\left(5.5-10^{5}\partial_{y}u\right)}^{2.3}{\left(5.0-20\omega \right)}^{3.4}\\ {\left(5.5+10^{5}|f+\zeta |\right)}^{2.4}e^{-11.8}-1.0 \end{array}$$ (2)

where *V_s_* is the vertical wind shear between 200 and 850 hPa, *u* is the zonal wind shear at 500 hPa, ω is the vertical pressure velocity at 500 hPa, *f* is the Coriolis parameter, and ζ is the relative vorticity at 850 hPa. All variables are evaluated in the standard unit.

### Seed propensity index

The SPI (*4*) is a proxy for TC seed frequency, defined as

$$\text{SPI}=-\omega \frac{1}{1+Z^{-1/\alpha }}$$ (3)

$$\text{where}\;Z=\frac{f+\zeta }{\sqrt{|\beta +\partial_{y}\zeta |U}}$$ (4)

The variable ω is the vertical pressure velocity at 500 hPa. We consider only the ascending vertical velocity (i.e., negative ω) and zero out the descending portion as per (*4*). The nondimensional parameter *Z* quantifies the environmental favorability for vortex spinup, which is a function of the Coriolis parameter *f* and its meridional gradient β, and the relative vorticity at 850 hPa ζ. The constant parameters *U* = 20 m s^−1^ and α = 0.69 are determined in (*14*).

All variables represent the climatological mean and are averaged over each month over multiple decades as well as those used in the DGPI. The averaging periods are year 101 to 200 for the CMIP6 preindustrial control experiments, year 101 to 150 for the CMIP6 abrupt 4xCO_2_ experiments (the first 100 years ignored), year 11 to 50 for the HiRAM with 50-km resolution (HiRAM-50 km) and AM2.5 with 50-km resolution (AM2.5-50 km) experiments, and year 11 to 30 for the AM2.5-25 km experiments (the first 10 years ignored). The SPI has the same units as ω but is commonly expressed as a nondimensional percentage change.

### Shortwave cloud feedback

The shortwave cloud feedback is calculated in two steps. The first step is to calculate the cloud radiative response in the shortwave spectrum between the preindustrial control and the 4xCO_2_ experiments using the kernel method (*31*). The cloud feedback is then the linear regression slope between the time series of the cloud radiative response and the global mean surface temperature change in the 4xCO_2_ experiment (*26*). Note that the cloud radiative response is averaged over the tropical Northern Hemisphere ocean, and the surface temperature is averaged globally, similar to the decomposition of (*9*). For the atmosphere-only models, the shortwave cloud feedback is estimated by the shortwave cloud radiative response between the control and the +2 K climates averaged over the statistically steady state, divided by the magnitude of the constant, prescribed SST increase (*32*).

### Effective climate sensitivity

The ECS is calculated from the abrupt 4xCO_2_ experiment, using the linear regression between the top-of-atmosphere energy imbalance and the global mean surface temperature change (*33*). Note that the *x* intercept is divided by two to obtain the standard ECS historically defined based on an abrupt 2xCO_2_ experiment. The ECS represents the final surface temperature if the climate system adjusts based on the rate in the first 150 years of simulation.

### Coupled model experiments

We analyze the preindustrial control and the abrupt CO_2_ quadrupling experiments in the CMIP6 (*13*). Models whose horizontal grid spacing is 200 km or coarser are ignored because the distribution of tropical convection is not well resolved. Monthly mean model outputs are used to compute the DGPI, SPI, shortwave cloud feedback, and ECS.

### Uniform warming experiments

The uniform warming experiments are conducted using three different atmospheric global models, including the HiRAM-50 km (*34*), AM2.5-50 km (*35*), and AM2.5-25 km (*3*). All three models have been used extensively for TC research and are known to generate realistic historical distributions. The control experiment is driven by the climatological SST averaged between 1986 and 2005 with repeated annual cycles. The uniform warming experiment is driven by the same SST time series plus 2 K uniformly. The same SST time series are prescribed to all models. The experiment setup is identical to that documented in (*4*).

### Green’s function experiments

The Green’s function is estimated from experiments driven by localized SST perturbation. The centers of the shape of the SST perturbations are shown in Fig. 4C. The experiments are conducted using the Atmospheric Model version 4 with 100-km resolution (*36*). The control experiment is driven by the climatological SST averaged between 1980 and 2014 with repeated annual cycles, and the perturbation experiments follow the setup of (*23*).

### TC tracking

TCs are tracked using an algorithm that identifies features in the climate model output that satisfy a number of criteria resembling a real-world TC. The algorithm searches six-hourly output of the surface pressure and wind speed, vorticity at 850 hPa, and mid-level temperature fields to identify warm core cyclones (*37*). The threshold values used for the HiRAM-50 km, AM2.5-50 km, and AM2.5-25 km models are documented in (*38*).
