# Supplementary material for: Dependence of tropical cyclone seeds and climate sensitivity on tropical cloud response
Source: Sci Adv. 2024 Sep 11;10(37):eadi2779. doi: 10.1126/sciadv.adi2779 (PMC11389784; doi:10.1126/sciadv.adi2779)
Supplement: Supplementary file 1 — Figs. S1 and S2 [file sciadv.adi2779_sm.pdf]

Science Advances
AAAS

Supplementary Materials for

Dependence of tropical cyclone seeds and climate sensitivity on tropical cloud response

Tsung-Lin Hsieh *et al.*

Corresponding author: Tsung-Lin Hsieh, hsiehtl@princeton.edu

Sci. Adv. **10**, eadi2779 (2024)
DOI: 10.1126/sciadv.adi2779

This PDF file includes:

Figs. S1 and S2

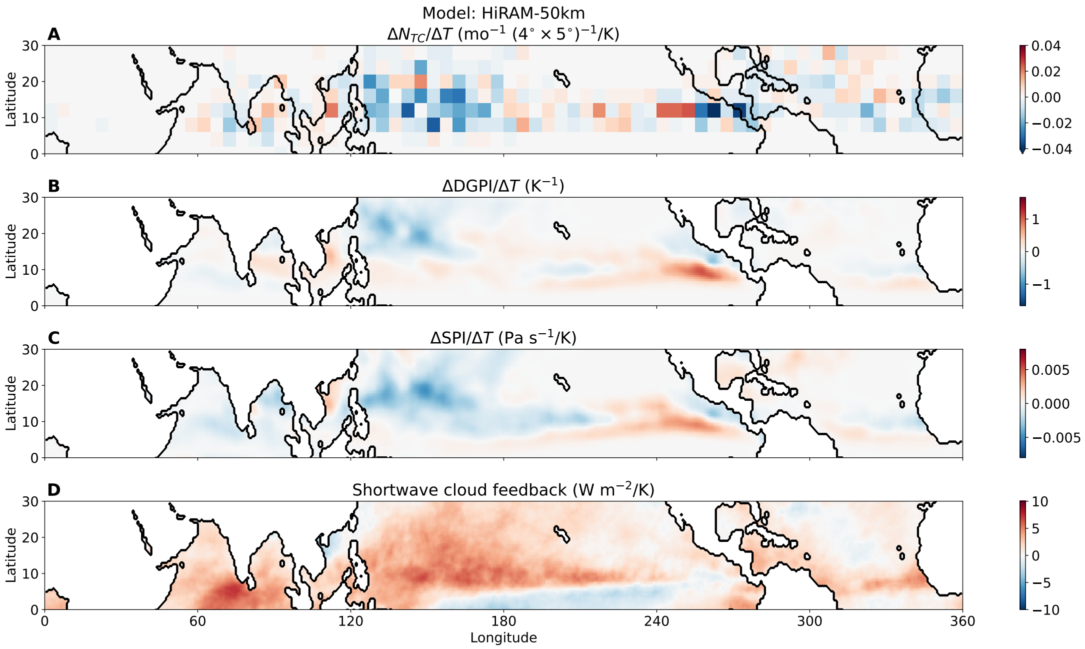

Figure S1: **Spatial distribution of response to uniform warming in HiRAM-50km.** As in Fig. 3, but for the HiRAM-50km model. (**A**) the change in TC frequency per Kelvin warming, (**B**) the change in DGPI per Kelvin warming, (**C**) the change in SPI per Kelvin warming, and (**D**) the shortwave cloud feedback, averaged from June to November.

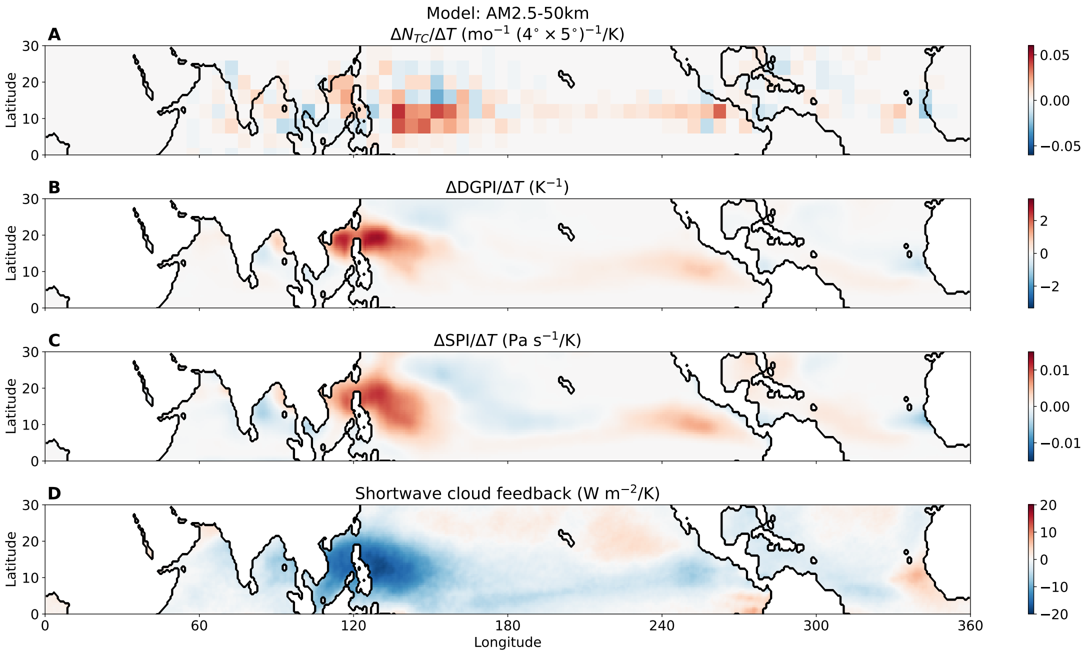

Figure S2: **Spatial distribution of response to uniform warming in AM2.5-50km.** As in Fig. S1, but for the AM2.5-50km model.
